# Oral pemphigus vulgaris after Chilean earthquake

**DOI:** 10.11604/pamj.2014.18.219.4273

**Published:** 2014-07-16

**Authors:** César Rivera, Bernardo Venegas

**Affiliations:** 1 Unit of Histology and Embryology, Department of Basic Biomedical Sciences, Faculty of Health Sciences, University of Talca, Talca, Chile; 2 Department of Oral Diagnosis, School of Dentistry, State University of Campinas (UNICAMP), Piracicaba, São Paulo, Brazil; 3 Mass Spectrometry Laboratory, Brazilian Biosciences National Laboratory - CNPEM, Campinas, São Paulo, Brazil; 4 Unit of Oral Pathology, Department of Stomatology, Faculty of Health Sciences, University of Talca, Talca, Chile

**Keywords:** Oral pemphigus vulgaris, earthquake, chile

## Image in medicine

A 62-year-old male, was referred for the management of extremely painful oral ulcerations with surrounding erythema on the soft palate and cheeks (A-C). He reported that ulcers began after the Chilean earthquake in 2010. His medical history was not significant. Ulcerations showed remissions and exacerbations since earthquake. On clinical examination, skin and other sites involvement was not found. An incisional biopsy was taken from the cheek region. Histopathological examination revealed suprabasilar blister formation associated with acantholysis (D and E). Hence, a final diagnosis of oral pemphigus vulgaris (OPV) was made based on histopathological pattern and clinical features. Treatment regimen included oral prophylaxis; prednisone and betamethasone 3-4 times daily for topical application. The patient was controlled every 2 weeks for the first 1 month. The lesions had diminished and increased healing with steroids within 4 weeks of starting the treatment. The patient was kept under observation for 12 months and the lesions showed signs of recurrence. Pemphigous vulgaris (PV) is a chronic inflammatory disease that affects the skin and the mucus membrane. It is a rare disease (0.1-0.5 cases/100,000 inhabitants/yr), with onset in the fifth or sixth decade of life. Oral lesions are the first manifestation in 50-90% of cases . PV results from an autoimmune process in which IgG antibodies are produced against normal desmosomal adhesion molecules (desmoglein 3, 1 and cadherin familly) on the cell membrane of keratinocytes. Recent evidence suggests that anxiety, depression, and psychological stress are correlated with the occurrence and intensity of associated OPV symptoms. OPV is a condition that has no cure. The response to treatment in oral lesions is much slower in comparison to cutaneous lesions.

**Figure 1:**
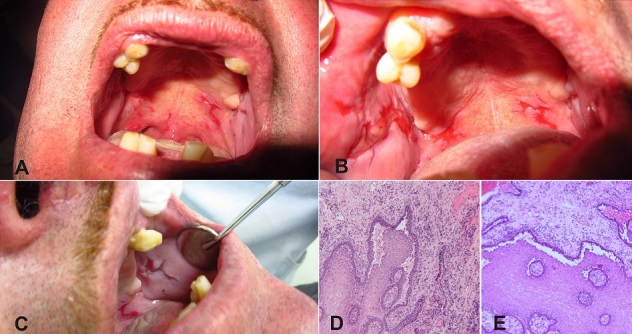
Asymmetric ulcerations with surrounding erythema on the patient's soft palate (A). Ulceration on the left cheek extended (B). Affected right cheek (C). Epithelial multilayered tissue with papillary hyperplasia. Suprabasilar separation (intraepithelial vesicle) which non adherent spinous cells floating in blister fluid (Tzanck cells)(D). The basal layer is seen attached to the underlying connective tissue without interrupts. (E) Connective tissue having an area of intense infiltrated inflammatory mononuclear and abundant vascular spaces with high congestion of erythrocytes (E).

